# Homosexual Fellatio: Erect Penis Licking between Male Bonin Flying Foxes *Pteropus pselaphon*

**DOI:** 10.1371/journal.pone.0166024

**Published:** 2016-11-08

**Authors:** Norimasa Sugita

**Affiliations:** 1 College of Science, Rikkyo University, Toshima, Tokyo, Japan; 2 Department of Botany, National Museum of Nature and Science, Tokyo, Tsukuba, Ibaraki, Japan; University of Lethbridge, CANADA

## Abstract

A recent focus of interest has been on the functional significance of genital licking (fellatio and cunnilingus) in relation to sexual selection in Pteropodid bats. In the present paper, a form of fellatio in wild Bonin flying foxes, *Pteropus pselaphon*, performed between adult males has been reported. During the mating season, adult flying foxes roost in same-sex groups, forming ball-shaped clusters which provide warmth. The female clusters may also contain a few males. Unassociated with allogrooming, same-sex genital licking occurred among males in the all male clusters. As such, male-male fellatio can be considered as homosexual behavior, two functional explanations could account for this behavior; the social bonding and the social tension regulation hypotheses suggested in a previous review. Given that neither the simpler alternative that in all male groups such fellatio may represent misdirected sexual behavior, nor the two previously proposed functional hypotheses were supported by the data, I propose another functional hypothesis. Homosexual fellatio in this species could help males solve inconsistent situations in the roost when there are conflicts between cooperative behavior for social thermoregulation and competition for mating.

## Introduction

Fellatio, the genital contact behavior by mouth of individuals on a male’s erect penis is widely used as foreplay during human copulation [1]. However, fellatio has been observed in other mammals between females and males, as well as same-sex individuals, both in captivity and in the wild [2]. Such same-sex fellatio is considered non-reproductive sexual behavior [2]. Tibetan macaque *Macaca thibetana*, males living in crowded male groups engaged in penis sucking of other males, which functioned as greeting and in reducing the tension between two males [3]. Juvenile male bonobos, *Pan panicus*, frequently fellate other juvenile males in the context of play [4]. In spotted hyenas, *Crocuta crocuta*, individuals of both sexes lick or sniff a male's erect penis and a female's erect pseudo-penis as a greeting ceremony in relation to social rank [5].

Recently, the genital licking of Pteropodid bats has attracted great attention as this group of bats offers a new functional explanation for the behavior. Female short-nosed fruit bats, *Cynopterus sphinx*, lick males’ erect penises during copulation (fellatio) and the duration of fellatio is positively related to the duration of copulation [6]. Males of the Indian flying fox, *Pteropus giganteus* lick the females’ vulva pre- and post-copulation (cunnilingus), and the duration of pre-copulation cunnilingus is correlated with the duration of copulation [7]. These two studies suggest that oral sex in Pteropodid bats prolongs the duration of copulation and does so by either increasing fertilization success [6] or improving the sperm competition of the males performing cunnilingus [7].

Homosexual behavior (or same-sex sexual behavior) is defined as courtship, mounting and genital contact and stimulation between same-sex individuals [8], and has been documented in a wide variety of animal taxa [9]. Homosexual behavior has received attention due to the apparent paradox against an assumed basic law of nature, that of Darwinian procreation, whereby individuals seek to maximize the number of offspring they produce and the frequency of their genes passed on to future generations [8]. However, the occurrence of same-sex pairing and individual preferences for same-sex individuals has been maintained in populations [9]. Many researchers have observed examples of homosexual behavior and provided various explanations for its adaptive significance. For example, homosexual behavior can serve as a form of social play, for the formation of social bonds, to diminish intrasexual conflict, for acquiring mating skills, to facilitate kin selection, for social tension regulation, and for alloparental care [2,8,9]. In addition, non-adaptive explanations have also been posited, such as mistaken identity, functionless by-product of adaptation, maladaptation [2,8,9]. Homosexual behavior influences the social dynamics of wild populations in some species, and might act as a selective pressure that shapes social, morphological, and behavioral evolution [9].

In the present study, I provide confirmation of male-male genital licking in wild, free-living, Bonin flying foxes *P*. *pselaphon*, in which only males lick the genital area of other males. In Livingstone’s flying foxes, *P*. *livingstonii*, zoo-reared males engage in mutual allogrooming accompanied by licking of erect penises [10]. However, it is difficult to discriminate between male-male genital licking and basic social contact through allogrooming. If genital licking between males is homosexual behavior, it is possible that the genital licking would occur independently of allogrooming. Therefore, I discuss whether the fellatio of *P*. *pselaphon* males can appropriately be treated as homosexual behavior.

I consider and test two functional explanations for this behavior: (i) the social bond hypothesis, and (ii) social tension regulation hypothesis (see review in [8]). The social bond hypothesis proposes that homosexual behavior provides psycho-physical rewards via pleasure and reinforces long-term relationships. The social tension regulation hypothesis proposes that same-sex behavior represents a type of conflict prevention management [8]. In addition, I consider and test the non-adaptive hypotheses that such behavior arises incidentally as either a byproduct of male-male allogrooming or as redirected sexual behavior among males without access to females. As none of the tested hypotheses fitted with the findings, I develop a new functional hypothesis suitable for further testing.

## Materials and Methods

### Study site and animals

The Bonin flying fox, *P*. *pselaphon*, is endemic to the Ogasawara Archipelago, Japan, at the northern limits of the distribution of the genus *Pteropus*. The population size of the flying foxes is very low, ca. 200 individuals [11]. The Japanese Government has designated *P*. *pselaphon* as a Japanese national treasure, and the International Union for Conservation of Nature has categorized them as a critically endangered species [12]. *P*. *pselaphon* is strictly protected by the national laws of Japan and international regulation. *P*. *pselaphon* eats several types of plant materials including fruits, nectar, and leaves [13,14]. *P*. *pselaphon* shows minimal sexual size dimorphism [15].

I conducted this study on Chichijima Island (27°05′N, 142°11′E), where *P*. *pselaphon* annually forms an arboreal colonial roost in winter from October to mid-May [16,17]. The colonial roost includes ca. 100 individuals and is located in secondary forest with a canopy height varying between 10 and 15 m [17]. The canopy where the bats roosted consisted primarily of Bishop wood, *Bischofia javanica*, and *Schima mertensiana*. The area in which the animals roosted was about 50 × 50 m in 2009. Roosting members of *P*. *pselaphon* were divided into three distinct groups: a male group, including adult males and several subadults; a female group, including mostly females and a few adult males; and a subadult group, including subadult individuals of both sexes. Each group also included some ball-shaped clusters in which more than two flying foxes roosted in direct physical contact with each other (S1 Fig) [16,17].

These ball-shaped clusters have not been observed in other species of the genus *Pteropus*. It has been proposed that the clustering behavior could be a behavioral adaptation to the cold winter weather in one of the most northern distribution areas of the genus, because the frequency of clustering individuals and the number of individuals in clusters has decreased with increasing ambient temperature [16]. The clustering behavior has also been associated with a possible from of polygyny [16]. Some female clusters also contained a single male as well as multiple females. A male was observed to exclude other males from the periphery of female clusters, allowing this individual to monopolize copulations with clustering females. Clusters in the female group were assembled at one or two roosting trees, whereas clusters in the male group were scattered around the female group [16,17].

### Observations

Observations were made on the male group and the female group within the winter colonial roost during the period of April 13–24th, 2009. Over a total of 8 days, 37 h 15 min (average of 4 h 39 min per day) was spent observing allogrooming and genital licking behavior using scan sampling with visual observation and binoculars (10 × 56 Nikon). Allogrooming behavior was defined as one individual licking the body of another individual, except the genital area. Genital licking behavior was defined as when an individual commenced genital licking events, including successive genital licking that was repeated at intervals of a few minutes until licking of the genial area ceased, when resting in a cluster or moving to another cluster, followed by additional copulation in the case of male–female pairs. A genital licking event was defined as one individual licking the genital area (penis and scrota on males, and vulva on females) of another individual. The direction in genital licking behavior was divided into four categories: (1) one male licking another male’s penis and scrota (male–male genital licking), (2) one male licking a female’s vulva (male-female genital licking), (3) female–female genital licking, and (4) female-male genital licking. The number of genital licking behavior was counted following the definitions above. The duration of genital licking behavior and allogrooming was not measured; therefore, one genital licking behavior could include multiple genital licking events by the same individuals. During copulation, including genital licking, mounting, ejaculation, and female refusal, males utter growling sounds, except when biting the nape of the females’ neck, and females utter successive shrieking sounds, as in *P*. *alecto* copulation [18,19]. I recorded the sounds made by Bonin flying fox males during same-sex genital licking behavior, to determine if they were similar to those made by males and females during copulation. I observed the number of allogrooming events in the same combinations as described above for licking sexual direction.

I also collected clear video and still photographs as evidence of genital licking behavior between males whenever possible. These behaviors were observed between 2004 and 2009 (except for 2005), during ecological investigations in the winter colonial roosts (DCR-TRV30 and HDR-SR12 Sony and D200 Nikon).

### Statistical analysis

The frequency of male–male and male-female genital licking behavior was analyzed using a generalized liner model (GLM) with R version 3.3.1 [20]. I used a GLM with Poisson errors and a log-link function, controlling for male population numbers of each group per day as an offset term. On average, there were 30.6 males (range: 22 to 45) in the male group and 4.6 males (range: 4 to 5) in the female group.

### Ethics statement

This study relied only on observational data and did not involve any capturing or handling of wild animals. Although Bonin flying foxes have been designed as an endangered species and are protected by both the government of Japan and the international community, no permission for observation was required. Permission for entry to the roosting area was obtained from the Finance Section, Ogasawara Village, Japan.

## Results

**Fig 1:**
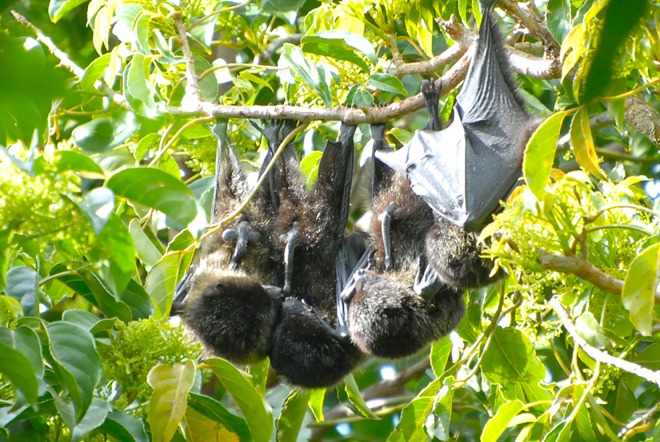
A still picture showing male–male genital licking (homosexual fellatio) of Bonin flying foxes. A male licked a neighbor male’s penis in a cluster including three individuals on February 15th, 2008.

Genital licking behaviors were photographed and filmed (Fig 1, S1 and S2 Videos). A total of 29 genital licking behaviors were observed in the winter colonial roost, eight of which were male–male (Fig 1) and 21 were male-to-female. All male-male genital licking occurred in the male group, and all male-female genital licking in the female group. Female–female and female-male genital licking were never observed. Male–male genital licking occurred at a lower frequency per day (1.0 ± 1.4 times, mean ± SD) than male-to-female genital licking (2.6 ± 2.4) (GLM: χ^2^ = 53.39, df = 1, P < 0.05).

Before male–male genital licking, the males rested in clusters or engaged in autogrooming while in clusters or alone. Licking males either moved from another cluster or participated in the same cluster with licked males. During autogrooming, males also licked their own penises and achieved erections. Immediately before male–male genital licking, both males autogroomed or awoke in a cluster including two focal males and more than one individual. Male–male genital licking events occurred during autogrooming (S1 and S2 Videos). One male licked the scrotum and shaft of another male. Male–male genital licking events occurred repeatedly several times in the same pair, and reciprocal genital also licking occurred (S1 Video). However, licking did not lead to ejaculation and mounting in all cases. No males licking the penises of other males emitted the growling sounds observed during heterosexual copulation. The sounds were recorded in all 21 copulations but not in 8 male-male genital licking behaviors. After male–male genital licking, the males continued to engage in autogrooming and either stayed in the cluster in which the genital licking occurred or moved to another cluster. Finally, the males rested.

All male-to-female genital licking (21 out of 21 times) occurred pre-copulation and then led to copulations. All males emitted growling sounds when attempting to lick the genital areas of females and mount the back of females. Some females moved to other clusters when a male attempted or began to lick their genital areas, to avoid copulation. When the males failed to copulate in the female group, they did not move to the male group to engage in male-male genital licking, but remained in the female group.

## Discussion

In the present study, I obtained positive evidence of male–male genital licking in *P*. *pselaphon* under natural conditions. Male–male mutual grooming has been observed in grey-headed flying foxes, *P*. *poliocephalus*, [21], and captive Livingstone’s flying foxes *P*. *livingstonii* engaged in allogrooming with genital licking between males [10]. However, such genital licking between *P*. *livingstonii* males may be a by-product of allogrooming as both behaviors occurred together. Allogrooming in *P*. *pselaphon* has not been previously observed, and was not observed in the present study. Therefore, the male-male genital licking in the Bonin flying foxes does not appear to be an infrequent by-product of allogrooming between males, but a behavior of directly licking the male genital area, independent of allogrooming. I consider genital licking between males to be a same-sex sexual (homosexual) behavior and defined as genital contact between individuals of the same sex [8].

In this study, there was insufficient observation time to determine whether the homosexual fellatio is a widespread or limited phenomenon within the population. Moreover, marked individuals were absent in the observational period, except for one radio-collared animal [16]. It is possible that this small number of males specifically engaged in male genital licking. However, male-male genital licking has been observed over five years of ecological investigations [16,17], including reciprocal genital licking between two male individuals (S1 Video), showing that multiple males engage in male–male genital licking. Although the sexual orientation of *P*. *pselaphon* individuals engaging in homosexual fellatio is unknown, it is unlikely that the phenomenon is driven by a few exclusively homosexual males.

Homosexual fellatio of *P*. *pselaphon* appears to be similar to heterosexual cunnilingus in that licking was limited to the genital area. Many *Pteropus* males lick female genital areas pre-copulation, including *P*. *poliocephalus* [21], Pacific flying foxes, *P*. *tonganus* [22], Black flying foxes, *P*. *alecto* [19], and Indian flying foxes *P*. *giganteus* [7]. If the homosexual fellatio of *Pteropus* males occurs in the mating context as in heterosexual cunnilingus, it is possible that homosexual fellatio could be performed to redirect the sexual frustrations among males in the male group where copulations were confined. However, in the homosexual fellatio of *P*. *pselaphon*, the males did not utter the loud sounds that were vocalized by males during copulation, begin to mount in the face-to-back intercourse position, nor ejaculate after genital licking, thus differentiating homosexual fellatio from the cunnilingus observed in a pre-copulation context. Thus, homosexual fellatio occurred in a context unrelated to mating that was described in the previous studies of the sexual activity in *Pteropus* species [7,18,21,22]. It is unlikely that homosexual fellatio conciliated sexual frustrations; therefore, it is possible that the function of homosexual fellatio in *P*. *pselaphon* could be explained by the high sociality in the roost, including sexual segregation and social thermoregulation, but not sexual arousal.

The functional significance of homosexual fellatio remains a mystery; however, many explanations for wide-spread same-sex behavior have been proposed, including social play, social bond formation, intrasexual conflict, practice for adult heterosexual copulation, kin selection, social tension regulation, alloparental care, and that it serves no function, mistaken identity, evolutionary by-product, and maladaptation [8,9]. Potential adaptive explanations of homosexual fellatio in *P*. *pselaphon* are linked to (i) the social bond and (ii) social tension regulation hypotheses [8] because of the existence of cooperation in forming clusters and competition among males for mating in the roost [16]. The social bond hypothesis proposes that homosexual behavior provides psycho-physical rewards via pleasure and reinforces long-term relationships [8,23]. Thus, homosexual fellatio would convey the intention to cooperate in forming a cluster between the fellatio actor and receiver. However, females did not engage in genital licking with other females when they participated in female clusters. Homosexual behavior may be sexually dimorphic in *P*. *pselaphon*, as in Japanese macaques *Macaca fuscata* [23] and *M*. *thibetana* [3]. The degree of pleasure and reward may differ between the sexes. The social tension regulation hypothesis proposes that homosexual behavior represents a type of conflict prevention management [8]. *P*. *pelaphon* males are generally assumed to be potential competitors for mating [16], and may use stimulation for currency when forming clusters. However, homosexual fellatio was never observed after fights between harem males and intruder males in the female group [16]. These two hypotheses for adaptive explanations of homosexual behavior are not mutually exclusive [8]. Indeed, a combination of the two hypotheses could provide a reasonable explanation that homosexual fellatio solves an inconsistent situation in the roost when there are conflicts between cooperative behavior for thermoregulation and competition for mating in roosts.

I propose a hypothesis that suggests homosexual fellatio promotes cooperation in forming clusters of males that are potential competitors. In the *P*. *pselaphon* population on Chichijima, located at one of the northern limits of the genus, males in the colony tend to repulse each other in order to monopolize mating with females in the female group, but the males are forced to cooperate to form ball-shaped clusters for warmth [16]. The homosexual fellatio, which never resulted in ejaculation, could serve as currency to facilitate potential competitors to participate in clusters via stimulation of the penis. Therefore, *P*. *pselaphon* males would be expected to increase the frequency of homosexual fellatio in conjunction with increasing energy requirements and metabolic rates. To evaluate this hypothesis, a sufficient number of marked individuals will be needed to accurately estimate the degree of social bonding between males in a long-term study. In addition, analysis of feces for the production of a detailed list of food plants eaten on the basis of their DNA and a thermal imaging camera would be useful to estimate the degree of income and expenditure of heat values.

Twenty-two bat species engage in homosexual behavior, which have been categorized into six groups: mutual homosexual grooming and licking, homosexual masturbation, homosexual play, homosexual mounting, coercive sex, and cross-species homosexual sex [24]. Two Pteropodid and one Phyllostomid bat species only engage in same-sex grooming and licking [10,21,24,25]. In the wild, *P*. *poliocephalus* engages in allogrooming, in which a male licks and gently bites the chest and wing membrane of the other, often with an erect penis [21]. In captivity, same-sex genital licking was reported among *P*. *livingstonii* males in a zoo [10], and among vampire bats, *Desmodus rotundus*, males in the laboratory [25]. Heterosexual genital licking in copulation has been extensively reported in Pteropodid bats [6,7,18,21,22]. Considering its prevalence, genital licking in copulation may be considered as a phylogenetic feature of Pteropodid bats, especially for the genus *Pteropus*. This phylogenetic behavioral characteristic could facilitate an emergence of homosexual fellatio in *P*. *pselaphon*. Furthermore, the expression of homosexual activity could increase under demographic conditions in skewed sex ratios and in multimale mating systems such as those in many primates [8,26]. Demographic conditions with sex segregation may promote homosexual fellatio of *P*. *pselaphon*. To provide further insight into the origin of homosexual fellatio of *P*. *pselaphon*, it will be necessary to consider both the evolutionary history and demographic context, in addition to alternative functional explanations.

## Supporting Information

S1 Fig A ball-shaped cluster of Bonin flying foxes (*Pteropus pselaphon*). (JPG)

S1 Video One digital video capture showing male-male genital licking (homosexual fellatio) of Bonin flying foxes. Two males mutually licked penises and scrotums in autogrooming on March 15th, 2004.
(MP4)

S2 Video One digital video capture showing homosexual fellatio of Bonin flying foxes. One male licked another male's penis on February 14th, 2009.
(MP4)
