# Supplementary figures and images for: Homosexual Fellatio: Erect Penis Licking between Male Bonin Flying Foxes Pteropus pselaphon
Source: PLoS One. 2016 Nov 8;11(11):e0166024. doi: 10.1371/journal.pone.0166024 (PMC5100941; doi:10.1371/journal.pone.0166024)

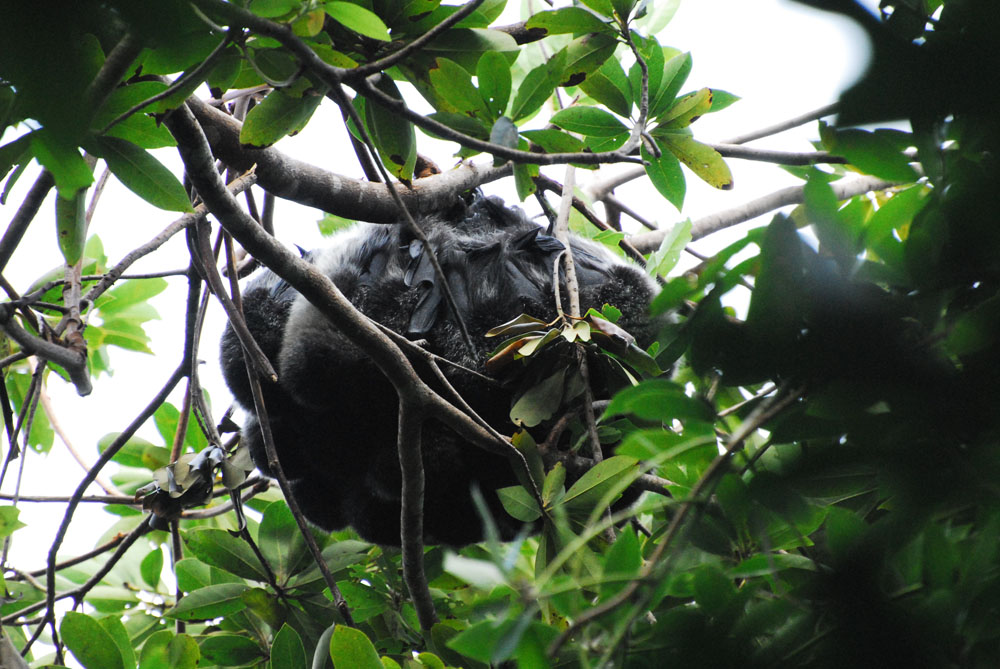

Supplement: S1 Fig — (JPG) [file pone.0166024.s001.JPG]
